# In-situ preparation of hierarchical flower-like TiO_2_/carbon nanostructures as fillers for polymer composites with enhanced dielectric properties

**DOI:** 10.1038/srep43970

**Published:** 2017-03-06

**Authors:** Nuoxin Xu, Qilong Zhang, Hui Yang, Yuting Xia, Yongchang Jiang

**Affiliations:** 1 School of Materials Science and Engineering, State Key Lab Silicon Mat, Zhejiang University, Hangzhou 310027, PR China

## Abstract

Novel three-dimensional hierarchical flower-like TiO_2_/carbon (TiO_2_/C) nanostructures were *in-situ* synthesized via a solvothermal method involving calcination of organic precursor under inert atmosphere. The composite films comprised of P (VDF-HFP) and as-prepared hierarchical flower-like TiO_2_/C were fabricated by a solution casting and hot-pressing approach. The results reveal that loading the fillers with a small amount of carbon is an effective way to improve the dielectric constant and suppress the dielectric loss. In addition, TiO_2_/C particles with higher carbon contents exhibit superiority in promoting the dielectric constants of composites when compared with their noncarbon counterparts. For instance, the highest dielectric constant (330.6) of the TiO_2_/C composites is 10 times over that of noncarbon-TiO_2_-filled ones at the same filler volume fraction, and 32 times over that of pristine P (VDF-HFP). The enhancement in the dielectric constant can be attributed to the formation of a large network, which is composed of local micro-capacitors with carbon particles as electrodes and TiO_2_ as the dielectric in between.

Advances in portable electronic devices, flexible electronic devices and electric power systems create a high demand for low-cost, compact, and efficient capacitors^1^,^2^,^3^. In the past decade, inorganic/polymer composites have exhibited tremendous potential for high-performance capacitors owing to their inherent merits of large dielectric constant (*ε*_*r*_), good processability and excellent flexibility^4^,^5^,^6^,^7^.

Recently, many efforts have been devoted to obtaining high-*ε*_*r*_ composites by introducing conductive fillers, including metal powders (e.g., silver, copper) and carbon-based materials (e.g., carbon black, graphene, carbon nanotubes), into the polymer matrix^8^,^9^,^10^,^11^,^12^,^13^,^14^. The existence of conductive components could give rise to the formation of a micro-capacitor network, and thus increases *ε*_*r*_. And the dielectric permittivity would rocket when the content of fillers approaches the percolation threshold. Nevertheless, the remarkable enhancement is always accompanied with ultra-high dielectric loss and the risk of insulator-conductor transformation owing to the continuous contact among conductive fillers^2^,^15^,^16^.

Another feasible approach is the incorporation of various high-dielectric-constant metal oxides^17^,^18^,^19^,^20^. Among the class of inorganic fillers, titanium dioxide, which features advantages such as low dielectric loss, corrosion stability, biological inertness and environmental benignity, has captured considerable attention^20^,^21^,^22^. However, a high volume fraction of fillers is usually required to achieve a high dielectric permittivity, which would inevitably deteriorate the flexibility of the composite film. Besides, the dielectric constants of TiO_2_-filled composites are usually lower compared with their ferroelectric-filled counterparts.

In order to abate the negative impacts in single-component incorporated composites, it is suggested that constructing ternary system containing both insulating and conductive fillers might be a good choice to obtain high-*ε*_*r*_materials at lower filler contents^6^. The main drawback of this approach is that the simple mixing process could not effectively prevent conductive particles from aggregation, which still worsens dielectric behaviors^5^. Recently, some attempts have been made by incorporating conductor/metal oxide heterostructures into polymer matrices. For example, Luo *et al*. reported that nano Ag-deposited BaTiO_3_ hybrid structure successfully suppressed the conductive pathway caused by agglomeration of conductors in the polyvinylidene fluoride (PVDF) composite^16^. As a result, the composites exhibited a high dielectric constant of 94.3 and a low conductivity with 43.4 vol.% filler loading at 1 kHz. Aepuru and Panda utilized carbon-decorated hollow radial ZnO (CZnO) as fillers in the fabrication of composites^23^. At a content of 35 wt.%, the dielectric permittivity of CZnO/PVDF was approximately fivefold compared with that of hollow radial ZnO-filled sample. Therefore, the rational design of filler microstructure plays a pivotal role in the dielectric properties of resultant composites.

In this study, we present a new strategy for fabricating high-*ε*_*r*_ polymer composites. Poly(vinylidene fluoride-co-hexafluoropropylene) [P(VDF-HFP)] is utilized as the matrix due to its relatively high dielectric constant (~10). Novel hierarchical flower-like TiO_2_/carbon (TiO_2_/C) structures are *in-situ* prepared via a solvothermal process, and carbon content is precisely adjusted by controlling thermal decomposition of organic precursor under inert atmosphere. The composite films comprised of P (VDF-HFP) and as-prepared hierarchical flower-like TiO_2_/C are fabricated by a solution casting and hot-pressing approach. The results reveal that the P (VDF-HFP)/flower-like TiO_2_/carbon nanocomposites lead to large network formation of local micro-capacitors and show enhanced dielectric properties.

## Results and Discussion

### Characterization of TiO_2_/carbon nanostructures

**Figure 1:**
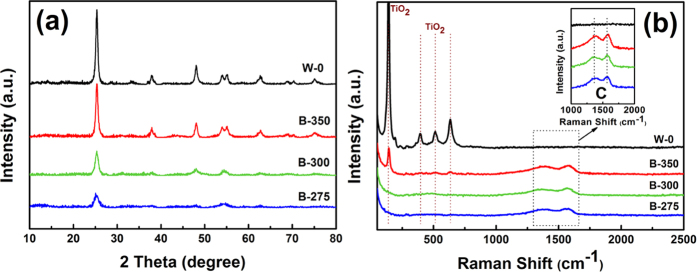
(**a**) XRD patterns and (**b**) Raman spectra of the powders calcined in air (W-0) and in argon atmosphere (B-275, B-300 and B-350).

XRD patterns of the powders after calcination under different conditions are presented in Fig. 1(a). The diffraction peaks of all samples could be indexed to the anatase TiO_2_ based on JCPDS card No. 21–1272. For samples calcined under argon atmosphere, no graphitic structure of carbon species could be detected, indicating that carbon might exist as amorphous phases. And the total intensity of TiO_2_ characteristic peaks increases gradually with increasing pre-calcination temperature. This phenomenon may be due to the interference of the carbon content in the three samples, which will be discussed later in this paper.

Raman spectroscopy is performed to further identify the phase composition of calcined particles. All Raman peaks of sample W-0 can be assigned as the A_1g_ (514 cm^−1^), B_1g_ (396 and 514 cm^−1^), and E_g_ (144, 196 and 635 cm^−1^) modes of the anatase phase, respectively^24^,^25^. The characteristic peaks of anatase phase subside and even vanish, and two modes around 1350 cm^−1^ and 1590 cm^−1^ appear in the spectra of B-275, B-300 and B-350. These two peaks are the fundamental D and G bands for carbon. The E_2g_ mode at 1590 cm^−1^ is associated with the sp^2^-bonded carbon atoms in a two-dimensional hexagonal graphitic layer. The peak at 1350 cm^−1^ corresponds to the existence of defects in the hexagonal graphitic layers^25^. The Raman spectra and XRD patterns confirm that B-275, B-300, B-350 are composed of anatase titanium dioxide and amorphous carbon. And sample W-0 exhibits a pure anatase phase.

**Figure 2:**
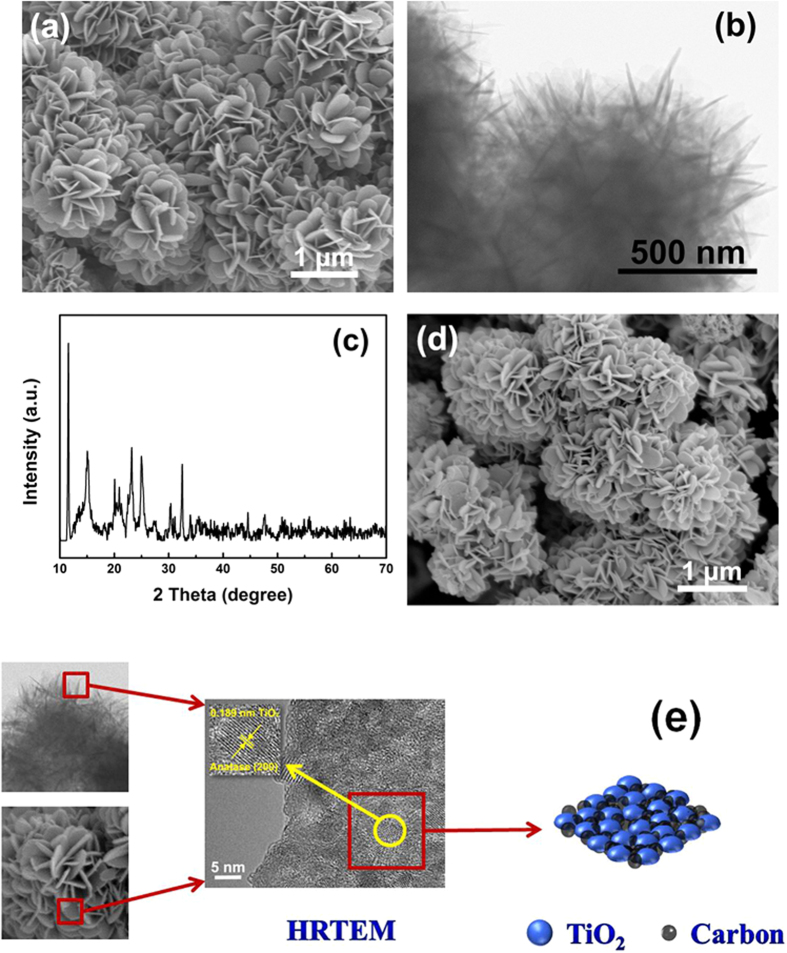
(**a**) SEM, (**b**) TEM images, and (**c**) XRD pattern of the solvothermal products; (**d**) SEM image, and (**e**) HRTEM image and structural schematic drawing of the calcined particles (B-300).

Figure 2(a) and (b) show the typical morphology of the solvothermal products, which are composed of 3D flower-like architectures with diameters ranging from 0.8 to 1.2 μm. Detailed observations reveal that the entire architecture is actually constructed from numerous 2D petal-like nanoflakes with smooth surfaces. The nanopetals are estimated to be around 10 nm in thickness, and connect with each other to form quasi-spheres. The XRD pattern of the precursor, as exhibited in Fig. 2(c), shows a strong peak located around 11° with several weaker ones at higher angles. Although the diffraction peaks couldn’t be matched to any known structure in the crystallographic databases, they are similar to those of reported Cu and In-based glycerolates^26^,^27^. Thus, we could deduce that the as-synthesized precursor is a titanium glycerolate. It is worth noting that the flower-like structures wouldn’t disintegrate into scattered nanoflakes even after calcination and prolonged ultra-sonication [Fig. 2(d)]. The stable architectures indicate that they are ordered self-assemblies^28^. Figure 2(e) illustrates the high-resolution TEM (HRTEM) image of the edge of the petal-like nanoflakes. The lattice fringes corresponding to anatase TiO_2_ can be observed, and the crystalline nanoparticles are surrounded by thin amorphous regions. Combining the XRD and Raman results, we could deduce that TiO_2_ nanoparticles are embedded among carbon layers, which are *in-situ* generated from the titanium glycerolate precursor.

**Figure 3:**
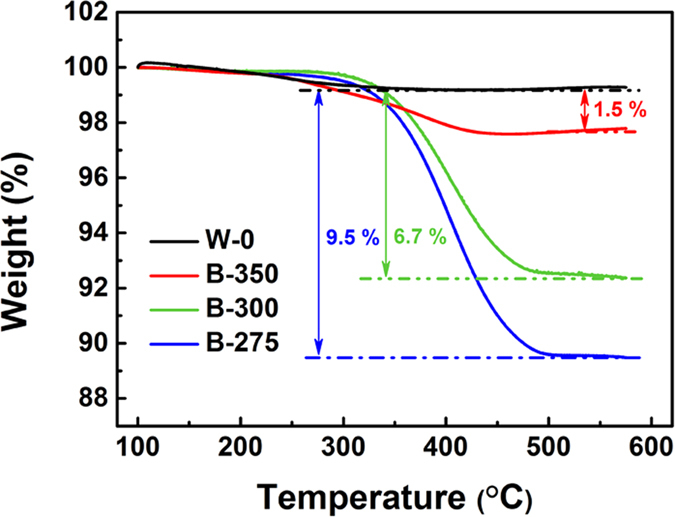
TG curves of the powders calcined in air (W-0) and in argon atmosphere (B-275, B-300 and B-350).

The carbon contents of the samples calcined in argon atmosphere (B-275, B-300 and B-350) are determined by TG analysis, as exhibited in Fig. 3. For comparison, the TG curve of W-0 is also presented here. Only one weight loss below 200 °C can be observed for pure TiO_2_, which could be attributed to the evaporation of absorbed gaseous or moisture contents. The TG curves of samples annealed in argon show two obvious mass loss stages. Besides one accompanied by a slight weight loss in the low temperatures, the other stage in the temperature range above 300 °C can be ascribed to the combustion of carbon^29^. According to the TG results, the carbon content of the black powders is about 1.5 wt.%, 6.7 wt.% and 9.5 wt.% for sample B-350, B-300 and B-275, respectively.

### Characterization of composites

**Figure 4:**
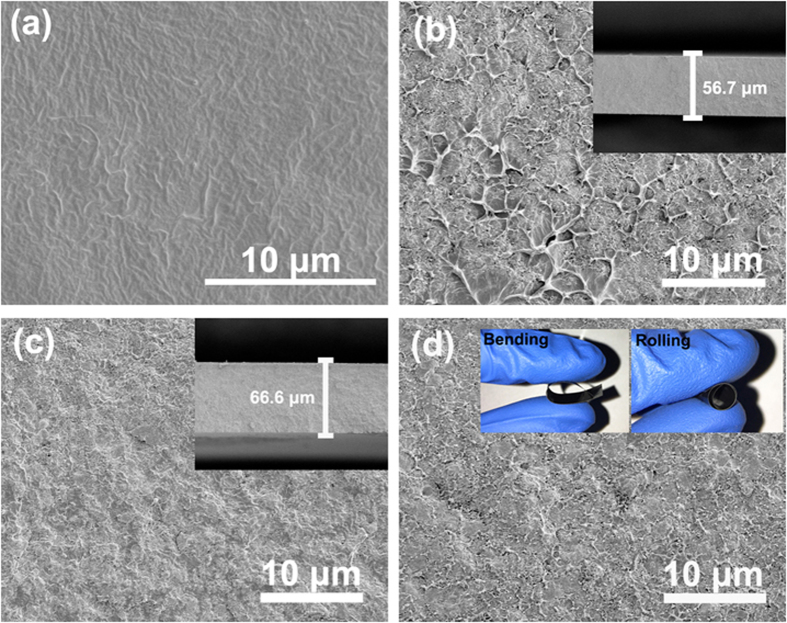
Cross-sectional SEM images of (**a**) pure P(VDF-HFP), and its composites filled with (**b**) 15 vol.% B-350, and 20 vol.% (**c**) B-300, (**d**) B-275. The insets in (**b**) and (**c**) are the typical film thicknesses. The insets in (**d**) are the digital photos of the sample filled with 20 vol.% B-275 particles, showing the flexibility of the film.

The SEM images of the cross-sections of pure P(VDF-HFP) and its composites are illustrated in Fig. 4. As shown in Fig. 4(b–d), TiO_2_/C fillers are homogenously dispersed in the matrix, and few voids could be observed between the particles and the polymer. Some shallow pits might be due to the freeze-fracture process. The phenomenon suggests that excellent adhesion is formed at the inorganic/organic interface, which is beneficial to the dielectric properties of the composites. In addition, the composite films exhibit good flexibility even at relatively higher filler contents, which can be demonstrated by the digital photos inserted in Fig. 4(d).

**Figure 5:**
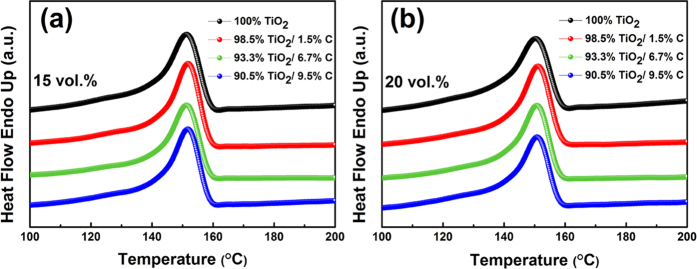
Heating curves of P(VDF-HFP) composites filled with (**a**) 15 vol.% and (**b**) 20 vol.% TiO_2_-based particles.

DSC analysis is adopted to investigate the effect of fillers on the crystallization behavior of the polymer matrix. The heating curves of composites filled with 15 vol.% and 20 vol.% TiO_2_ or TiO_2_/C particles are shown in Fig. 5(a) and (b), respectively. As demonstrated in Fig. 5, each sample exhibits only one single melting peak, whose position is similar to those of other composites. The crystallinity (*χ*_*c*_) of the polymer is calculated using the following equation^30^:


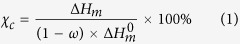


where Δ*H*_*m*_ is the heat enthalpy of the sample, 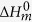 is the heat of fusion for the perfect P(VDF-HFP) crystal (93.07 J/g), and *ω* is the weight percentage of TiO_2_ or TiO_2_/C particles in the matrix. The crystallinities of polymer in different samples are calculated and summarized, as shown in Table 1. In general, the crystallinities of the composites are all lower than that of the pristine polymer. The crystalline fraction is enhanced at a low loading level of carbon. For instance, *χ*_*c*_ varies from 35.43% for TiO_2_-P (VDF-HFP) to 37.33% for the composite filled with 15 vol.% B-350 particles. However, the degree of crystallinity declines as the carbon loading further increases. This phenomenon could be attributed to the combined effect of different mechanisms^31^. On one hand, the incorporation of carbon creates more heterogeneous nucleation sites, and thus decreasing nucleation energy and enhancing the crystallization of the polymer matrix. On the other hand, the introduction of loading particles sets up physical obstacles and the motions of the polymer chain are then hindered.

### Dielectric properties of composites

**Figure 6:**
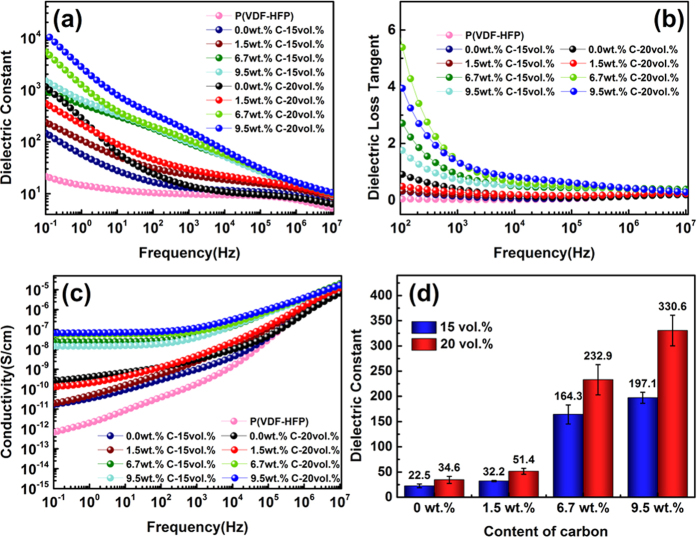
Frequency dependence of (**a**) dielectric constant, (**b**) dielectric loss, (**c**) conductivity, and (**d**) variation of dielectric constant as a function of the carbon content at 100 Hz of P(VDF-HFP) and its composites. The indicated error bars shown are the standard deviations of multiple dielectric constants.

Figure 6(a–c) present the variations of the dielectric constant (*ε*_*r*_), dielectric loss tangent (*tan δ*) and electrical conductivity (*σ*) of P(VDF-HFP) and its composites as a function of frequency with varied filler volume fractions and carbon loadings at room temperature. As illustrated in Fig. 6(a), all composites exhibit increased dielectric constants compared with those of pristine P(VDF-HFP). For pure P (VDF-HFP) and composites incorporated with TiO_2_ particles, the dielectric constant declines with increasing frequency in the low-frequency region. And the frequency dependence becomes stronger with the increase of the loading of TiO_2_ in the composites. At high frequencies, the variation trend of *ε*_*r*_ becomes relatively stable. In the case of composites filled with low-carbon-content TiO_2_/C particles, the frequency dependence is similar to that of noncarbon-TiO_2_-filled ones. The decrease of the dielectric constant with the frequency shows the typical characteristic of interface polarization. The interfacial polarization is also known as Maxwell-Wagner-Sillars (MWS) polarization, which occurs when there is an accumulation of charge carriers at the interfaces of heterogeneous systems. The MWS polarization only makes contributions at low-frequency range due to its long relaxation time, therefore the variation of *ε*_*r*_ becomes steady at higher frequencies^19^. When the carbon loading within the fillers becomes higher, the dielectric constant decreases with the frequency over the whole measurement range. Similar trends have been reported in literature for other polymer composites containing conductive fillers^2^,^13^. In order to make a comparative analysis, the dielectric constants at 100 Hz of the composites are extracted and given in Fig. 6(d). It is obvious that the TiO_2_/C-P (VDF-HFP) composite samples exhibit much higher dielectric constants in comparison with TiO_2_-filled ones. And *ε*_*r*_ increases with the increase of carbon content. Typically, the highest dielectric constant (330.6) of the TiO_2_/C composites is 10 times over that of noncarbon-TiO_2_-filled composites at the same filler volume fraction, and 32 times over that of pristine P(VDF-HFP). The enhancement in dielectric constant can be mainly attributed to the gradual formation of the micro-capacitor network as the content of conductive carbon increases^32^,^33^. Each two neighboring carbon particles are treated as a local capacitor with the C particles as electrodes and TiO_2_ as the dielectric in between. And a large network of these local micro-capacitors is constructed between two macroscopic electrodes, resulting in the significant promotion of the dielectric permittivity.

The differences in dielectric loss tangent and conductivity among the P (VDF-HFP) composites are also closely related to the content of carbon within the fillers. As exhibited in Fig. 6(b) and (c), the composite filled by inclusions with larger loading level of carbon shows not only higher dielectric loss, but also higher conductivity, and the dielectric loss tangent exhibits a sharp decreasing trend at low frequencies. The decline could be explained by the existence of extra sources of space charge carriers in the system, which is induced by the inorganic fillers^34^. It is worthy to be mentioned that composites filled with B-350 (i.e. 1.5 wt.% carbon-loading TiO_2_/C) particles exhibit lower loss tangents compared with W-0 (i.e. noncarbon-TiO_2_) filled ones. For instance, the loss tangent of the P(VDF-HFP) composite filled with 20 vol.% B-350 is 0.50 at 100 Hz, in contrast to 0.94 for the composite filled with 20 vol.% W-0. Meanwhile, the dielectric constant of B-350-incorporated sample is 51.4, which is 1.5 times over that of the W-0-incorporated one. The results imply that loading the fillers with a small amount of carbon is an effective way to improve the dielectric constant and suppress the dielectric loss. The conductivity increases with frequency for the composites with low-carbon-content and noncarbon fillers owing to the insulating nature. However, the plots for the composites filled with high-carbon particles exhibit a plateau at low frequencies, then the plateau starts to bend upwards at about 100 Hz and the conductivity becomes proportional to frequencies afterwards. The following theory might interpret this variation trend^35^,^36^,^37^. In the low frequency plateau region, the charge carriers can transport over a long distance. The conductivity is mainly determined by local percolating paths, which are formed as the content of carbon increases. Therefore, the conductivity is independent of frequency. When the frequency varies up to around 100 Hz, the contribution of the capacitors becomes dominant, and thus the composites exhibit typical frequency-dependent behaviors of dielectric materials.

**Figure 7:**
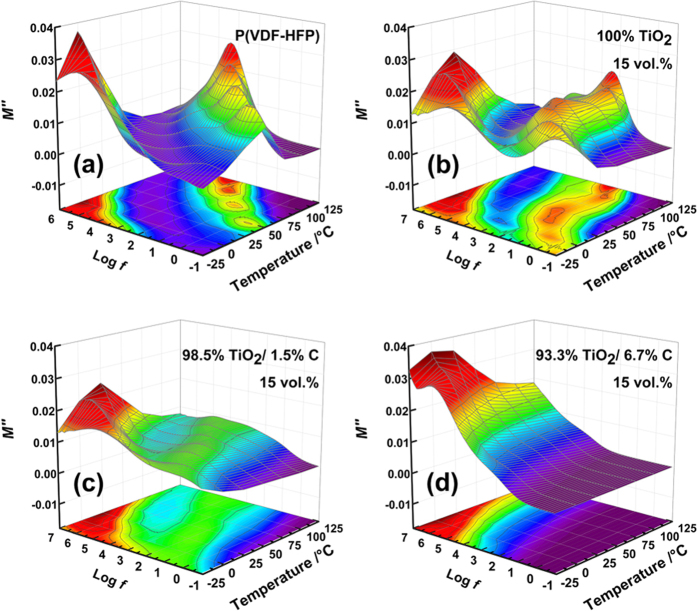
Frequency dependence of the imaginary part of electric modulus at various temperatures of (**a**) P(VDF-HFP), and its composites filled with 15 vol.% (**b**) TiO_2_, (**c**) 1.5 wt.% carbon-loading TiO_2_/C and (**d**) 6.7 wt.% carbon-loading TiO_2_/C particles.

In order to obtain more information about the dielectric relaxation behaviors of the composites, we analyze our experimental data using the electric modulus formalism. The electric modulus formalism is independent of parasitic effects (e.g. space charge injection, absorbed impurity conduction) and reflects the relaxation existing in different energy environments. The complex electric modulus formalism M^*^ is defined by the following formula^38^:


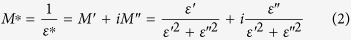


where M′ and M″ are the real and the imaginary part of electric modulus, respectively, and ε′ and ε″ are the real and the imaginary part of dielectric constant. To evaluate the relaxation phenomena within the bulk materials, the imaginary part of the electric modulus (M″) is extracted, as displayed in Fig. 7. The obvious peaks in the high frequency range exhibit the α_a_ relax_a_tion, which originates from the segmental movement of polymer chains in the amorphous zone of the matrix^39^. At the low frequency side, there exists another relaxation process for all the samples, which should be attributed to the MWS_P(VDF-HFP)_ polarization. This polarization is caused by charge accumulation at the crystal/amorphous boundaries in P(VDF-HFP)^40^,^41^. In general, the peaks shift towards higher frequencies with the increase of temperature and carbon loading within the fillers. This phenomenon is due to the enhancement of mobility of charge carriers with the increase of temperature and conductivity, both of which are accompanied by the reduction of the relaxation time. Besides, the relaxation intensity of MWS_P(VDF-HFP)_ polarization subsides with the increase of carbon content, implying that carbon can help to suppress blocked charges at the boundaries between amorphous and crystalline regions.

## Conclusions

In summary, hierarchical flower-like TiO_2_/carbon (TiO_2_/C) structures with various concentrations of carbon have been successfully prepared by controlled thermal decomposition of organic precursor under inert atmosphere. The microstructures of TiO_2_/C particles and their influence on the crystalline structure and dielectric properties of the corresponding composites have been investigated in detail. The experimental data imply that loading the fillers with a small amount of carbon is an effective way to improve the dielectric constant and suppress the dielectric loss. With the increase of carbon content, TiO_2_/carbon-filled composites show much greater dielectric constants in comparison with their noncarbon-TiO_2_-filled counterparts. Typically, the highest dielectric constant (330.6) of the TiO_2_/C composites is 10 times over that of noncarbon-TiO_2_-filled composites at the same filler volume fraction, and 32 times over that of pristine P(VDF-HFP). The enhancement in the dielectric constant can be mainly attributed to the formation of micro-capacitor networks, which are composed of carbon particles as electrodes and TiO_2_ as the dielectric in between. This work can provide a feasible strategy for fabricating flexible composites with high dielectric performance for integrated capacitors.

## Methods

### Chemicals and materials

Glycerol and ethanol were purchased from Sinopharm Chemical Reagent Co., Ltd., China. Titanium oxysulfate (TiOSO_4_) solution and poly(vinylidene fluoride-co-hexafluoropropylene) [P(VDF-HFP)] pellets were provided by Sigma-Aldrich. N, N-dimethyl formamide (DMF) was analytical grade and supplied by Aladdin Industrial Corporation, China. All chemicals were used as received without further purification.

### Synthesis of flower-like TiO_2_/carbon nanostructures

The flower-like nanostructures were prepared via a template-free solvothermal approach. In a typical synthetic procedure, A clear solution containing TiOSO_4_, glycerol and ethanol (molar ratio = 1:125:275) was stirred for 40 min at room temperature. The mixture was then transferred into a 100 mL Teflon-lined stainless steel autoclave, sealed and maintained at 110 °C for 48 h. After natural cooling down, the resultant precipitates were centrifuged, washed thoroughly with distilled water and ethanol and dried at 60 °C for 12 h. Afterwards the white powders were heated to 275–350 °C in air and maintained at the temperature for 1 h. Finally, the products were calcined under a flow of argon at 575 °C for 2 h. According to their pre-calcination temperatures, the obtained black samples were denoted as B-275, B-300, B-350, respectively. Meanwhile, some solvothermal products were calcined in air at 575 °C for 2 h. The white powders were named as W-0.

### Fabrication of P (VDF-HFP) composites

P (VDF-HFP) pellets were first dissolved in DMF and stirred for 4 h at room temperature. Then a designed amount of TiO_2_/C was ultrasonically dispersed into the solution for 30 min. And the suspension was vigorously stirred for 24 h to make it stable and homogenous. After that, the mixture was cast onto a clean glass plate by a piece of laboratory casting equipment (MSK-AFA-III, Hefei Ke Jing Materials Technology Co., Ltd.) and dried at 60 °C for 12 h to remove the residual DMF. Finally, the obtained composite films were molded by hot-pressing at 180 °C for 10 min under a pressure of 1500 psi. Pristine P (VDF-HFP) film was also prepared for comparison via similar procedures.

### Characterization

The phase composition of samples was identified by X-ray diffraction (XRD, EMPYREAN, PANalytical Co., Netherlands) analysis, using Cu K_α_ radiation in the 2θ range of 10°–80°. The morphology of the particles was observed using a field emission scanning electron microscope (FESEM, S-4800, Hitachi Ltd., Japan). Transmission electron microscopy (TEM) images were obtained from a CM 200 instrument operated at an accelerating voltage of 160 kV. Raman spectra were obtained using a DXR Smart Raman Spectrometer (Thermo Fisher, USA). Thermogravimetric analysis (TGA) was performed using a Perkin-Elmer Pyris 1 instrument between 100 °C and 550 °C with a heating rate of 10 °C min^−1^ in air. In order to obtain the cross-section morphology of the films, all the samples were freeze-fractured in liquid-nitrogen and then observed using a field emission scanning electron microscope (FESEM, SU-70, Hitachi Ltd., Japan). Differential scanning calorimetry (DSC) was conducted on a Perkin-Elmer DSC-7 analyzer at a heating/cooling rate of 10 °C min^−1^ in the temperature range from 80 °C to 2000 °C under a nitrogen flow. Copper electrodes of 150 nm in thickness were evaporated on both surfaces of the films using a mask with 20 mm diameter eyelets for the electrical measurements. Dielectric spectra were acquired using a Novocontrol Alpha-N high resolution Dielectric Analyzer (GmbH Concept 40) in the frequency range of 10^−1^ to 10^7^ Hz at several temperatures between −25 °C and 125 °C.

## Additional Information

**How to cite this article:** Xu, N. *et al*. In-situ preparation of hierarchical flower-like TiO_2_/carbon nanostructures as fillers for polymer composites with enhanced dielectric properties. *Sci. Rep.* **7**, 43970; doi: 10.1038/srep43970 (2017).

**Publisher's note:** Springer Nature remains neutral with regard to jurisdictional claims in published maps and institutional affiliations.

## Figures and Tables

**Table 1:** Evolution of the crystallinity *χ*_*c*_ of P(VDF-HFP) and its composites filled with TiO_2_-based particles.

		P(VDF-HFP)	TiO_2_	98.5% TiO_2_/1.5% C	93.3% TiO_2_/6.7% C	90.5%TiO_2_/9.5% C
Sample						
*χ*_*c*_/%	15 vol.%	41.37	35.43	37.33	35.08	34.16
	20 vol.%		32.32	34.74	31.32	30.93
